# Repeated Low-Dose Acrolein Triggers Irreversible Lamina Propria Edema in Urinary Bladder, Transient Voiding Behavior and Widening of Eyes to Mechanical Stimuli

**DOI:** 10.3390/cells10123477

**Published:** 2021-12-09

**Authors:** Sanghee Lee, Jiwoo Lee, Chandresh Khimji, Jaebeom Lee, Shelle Malkmus, Michael Albo, Tony Yaksh, George Chiang

**Affiliations:** 1 Department of Urology, University of California San Diego, San Diego, CA 92093, USA; salee@health.ucsd.edu (S.L.); jil963@health.ucsd.edu (J.L.); channykhim98@gmail.com (C.K.); jaebum1107@gmail.com (J.L.); malbo@health.ucsd.edu (M.A.); 2 Pediatric Urology, Rady Children’s Hospital San Diego (RCHSD), San Diego, CA 92129, USA; 3 Department of Anesthesiology and Pharmacology, University of California San Diego, San Diego, CA 92093, USA; smalkmus@health.ucsd.edu (S.M.); tyaksh@health.ucsd.edu (T.Y.)

**Keywords:** acrolein, cyclophosphamide (CYP), repeated low-dose acrolein model, urinary bladder, voiding dysfunction

## Abstract

Acrolein is a metabolite of cyclophosphamide (CYP), an alkylating agent used for a wide range of benign and malignant diseases. CYP treatments are known to trigger hemorrhagic cystitis in patients and animals. Significant effort has been made to prevent CYP/acrolein-induced cystitis, while still maintaining its therapeutic benefits. As a result, supplementary therapeutic options to mediate the protective role against CYP/acrolein and lower doses of CYP are currently given to targeted patients, as compared to past treatments. There is still a need to further study the effects of the repeated low-dose CYP/acrolein on the pathophysiology of the urinary bladder. In our study, a one-time treatment of acrolein and repeated low-dose acrolein triggered the thickening of the smooth muscle and lamina propria in the urinary bladder of C57BL/6J mice, respectively. The first dose of acrolein did not trigger voiding dysfunction, but the second dose triggered high-volume low-frequency voiding. Interestingly, our new scoring criteria and concurrent behavioral assessment revealed that mice with repeated low-dose acrolein had a wider opening of eyes in response to mechanical stimuli. Our study suggests that clinical symptoms among patients undergoing prolonged low-dose CYP may differ from previously reported symptoms of CYP-induced hemorrhagic cystitis.

## 1. Introduction

The alkylating agent cyclophosphamide (CYP) is a therapeutic option used for the treatment of certain non-neoplastic and a wide range of malignant diseases [1,2,3]. Upon intravenous or oral intake, CYP is metabolized to phosphoramide mustard and acrolein residing in targets cells and urinary bladder, respectively [4]. The mustard components inhibit cell replication by forming a covalent linkage with the nucleic acids of DNA. In parallel with its intended therapeutic effect, the urinary bladder is at most risk of CYP-induced urothelial cell damage due to the nature that acrolein, a metabolite of CYP, is a cytotoxic component stored in the urinary bladder until urine is voided. CYP/acrolein-induced urological complications include hemorrhagic cystitis, hematuria, azoospermia, vesicoureteral reflex, microhematuria, bladder cancer, etc. [5,6,7,8,9]. In 1988, Stillwell et al. reported that 100 patients with CYP (average dose of 4600 cGy)-induced hemorrhagic cystitis developed gross hematuria (78%), irritative voiding symptoms (45%), microhematuria (93%) and bladder cancer (5%) [4]. In that report, 56 patients out of 100 had persistent symptoms of CYP-induced hemorrhagic cystitis even after CYP treatment was stopped. Unveiling CYP-induced side effects including hemorrhagic cystitis influenced clinical and basic research communities to dedicate significant efforts to establish a therapeutic strategy to prevent CYP/acrolein-induced cystitis whilst maintaining the benefits of CYP. For example, mesna (2-mercaptoethanesulfonic acid) is given to patients and murine models that received CYP treatments resulting in a significant decrease in the incidence of hemorrhagic cystitis and its secondary effect [10,11,12]. Briefly, a sulfhydryl compound of mesna binds to acrolein, mediating a protective role against CYP/acrolein-mediated urothelial damage [13,14]. However, patients that have had an allergic reaction tomesna have been reported [15] and a need to develop alternative therapeutic options such as anti-vascular endothelial growth factor (VEGF) has been suggested [16,17]. In parallel with and in addition to a mesna supplement, the administration of a lower dose of CYP has been considered, which results in a decreased incidence of CYP-induced hemorrhagic cystitis [18]. Compared to comprehensive studies about the role of the conventional dosing of CYP on the urinary bladder, little is known about the effects of a repeated “low-dose” CYP/acrolein on the histopathology and physiology of the urinary bladder. Taken together, continuous efforts to develop uro-protective drugs against CYP and understand the role of repeated low-dose CYP/acrolein are required. In this study, we established a murine model recapitulating in vivo changes in the urinary bladder among patients that have received a low dose of CYP. The goal of this study was to focus on behavior changes in mice repeatedly taking low-dose acrolein.

## 2. Materials and Methods

### 2.1. Murine Models

The study protocol was approved by and performed in strict accordance with the Institutional Animal Care and Use Committee (IACUC) of the University of California San Diego.

### 2.2. A Murine Model of Acrolein-Induced Acute Cystitis

To demonstrate the feasibility of the study design, one dose of 85 µg of acrolein in 15 µL phosphate-buffered saline (PBS) was given to adult female C57BL/6 mice by transurethral instillation (acute cystitis model, *n* = 4). One dose of 15 µL PBS was given to the control group (*n* = 4). The body weight was measured on 4 and 24 h (hours) post instillation (pi).

### 2.3. Repeated Low-Dose Acrolein Model

Three doses of 10 µg acrolein in 15 µL PBS (repeated low-dose acrolein model, *n* = 9) vs. 15 µL PBS (control group, *n* = 8) were transurethrally given to adult female C57BL/6 mice on days 0, 7 and 14. Body weight was measured and voiding behavior was determined by filter paper assay (FPA) as previously described [19,20] on days 1, 8, 15 and 30 pi. Thus, mice in the acrolein group had a seven-day treatment interval in between the three doses of 10 µg acrolein, and a fourteen-day treatment-free period between the last dose of acrolein treatment and test/tissue harvest. The filter papers were imaged by BIORAD Gel Doc XR Imaging system (Bio-Rad Laboratories, Hercules, CA) and analyzed by counting numbers and measuring the area of urine spot for the determination of urinary frequency and voided volume. To further analyze the data from FPA, cut-off points for the voiding frequency and voided volume were established based on the 90th, 75th, 25th and 10th percentiles of mice studied as previously shown [19]. FPA was performed by observers blinded to the treatment conditions after the placement of animals in behavioral test units were randomized by another investigator.

#### 2.3.1. Histopathology

Histopathology of the urinary bladder of a murine model of acrolein-induced acute cystitis and its control group at 24 h pi was determined by hematoxylin and eosin (H&E) staining of formalin-fixed paraffin-embedded (FFPE) tissue slides sectioned with 5 µm thickness. The timeline for histopathology in the acute cystitis model was determined by previous publications that histopathological changes were shown as early as 4 h pi [21]. Histopathology of a urinary bladder from the repeated low-dose acrolein model and its control group at days 1 and 30 pi was determined by H&E staining.

#### 2.3.2. Behavioral Assessment

All behavioral tests were performed by observers blinded to the treatment conditions after the placements of the animals in behavioral test units were randomized by another investigator. To determine the role of repeated low-dose acrolein on scored symptoms, we adapted six different assessments: (1) abdominal withdrawal response to tactile stimulation; (2) tactile allodynia of hind paw; (3) grimace score; (4) daily parameter; (5) concurrent determination of abdominal withdrawal response to tactile stimulation and grimace score; and (6) the concurrent determination of the tactile allodynia of the hind paw and grimace score. The first three behavioral assessments were widely adapted in the field [22,23]. The fourth behavioral assessment was introduced by a previous publication [24]. The last two behavioral assessments were newly developed by this study. These assessments were each processed at baseline and on days 1, 8, 15 and 30 pi for the mice from the repeated low-dose acrolein model.

Behavioral Assessment 1—abdominal withdrawal response. Sensitivity of lower pelvic area (abdominal withdrawal response) in response to the selected von Frey filaments with a target force of 1.65, 2.44, 3.84, 4.93 and 5.88, was determined. Each filament was applied 10 times (10-times assessment of von Frey filaments to pelvic area) with a minimum 5 min interval time between the applications of a von Frey filament.

Behavioral Assessment 2—tactile allodynia. The fifth behavioral assessment was to determine the tactile allodynia of the hind paw by the von Frey filaments through Dixon’s up–down method. The von Frey filaments with a target force of 1.65, 2.44, 3.22, 3.84, 4.17, 4.56 and 4.93 were used. The 50% probability of withdrawal threshold was calculated as previously described [25,26].

Behavioral Assessment 3—grimace score. The third behavioral assessment was to determine a facial expression of pain by the grimace scale without any experimental stimulation. The five categories of facial expression of pain (orbital tightening, nose bulge, cheek bulge, ear position and whisker change) were scored based on the following scale: 0 = not present; 1 = moderately present; and 2 = obviously present as previously described [22].

Behavioral Assessment 4—daily parameter. The fourth behavioral assessment was to evaluate the behavioral status of (1) placing and stepping; (2) righting reflex; (3) spinal symmetry; (4) symmetric ablation; (4) lean; (5) hunched back; (6) catalepsy; (7) tremor; and (8) seizures by using the daily parameter score system. The daily parameter score was assigned based on the following scale: 0 = absence of change, normal behavior (baseline); 1 = slight change, noticeable change in parameter being evaluated, 5–25% change (occasional); 2 = moderate change, significant difference apparent upon first observation, 26–50% change (frequent); and 3 = marked or severe change, serious problems, >50% change (constant) as previously shown [24].

Behavioral Assessment 5—concurrent determination of abdominal withdrawal response to tactile stimulation and grimace score. The second behavioral assessment was to determine both the abdominal withdrawal response to tactile stimulation and the severity of pain felt by mice using a concurrent assessment of von Frey filaments as described above and the orbital tightening of the grimace scale, respectively. These concurrent assessments were completed by two observers. The orbital tightening of the grimace scale was scored based on the following scale: 0 = not present; 1 = moderately present; and 2 = obviously present. While we performed this concurrent assessment, we identified a new behavioral phenotype of the widening of eyes upon the von Frey filament application which was not previously recognized by the grimace score system and this was scored as an asterisk (*).

Behavioral Assessment 6—concurrent determination of tactile allodynia of hind paw and grimace score. The sixth behavioral assessment was to determine both the tactile allodynia of the hind paw and the severity of pain felt by mice using a concurrent assessment of the von Frey filaments by Dixon’s up–down method and the orbital tightening of the grimace scale, respectively. These concurrent assessments were completed by two observers. For the up–down method, the von Frey filaments that had a target force of 1.65, 2.44, 3.22, 3.84, 4.17, 4.56 and 4.93 were used. The 50% probability of withdrawal threshold was calculated as previously described [25,26]. Orbital tightening of the grimace scale was scored based on the following scale: 0 = not present; 1 = moderately present; and 2 = obviously present. A behavioral phenotype of widening eyes upon von Frey filament application, which was not previously recognized by the grimace score system, was scored as an asterisk *.

#### 2.3.3. Statistical Analysis

All data are presented as mean ± SE. Results were statistically analyzed using a paired *t*-test to compare the parameters from the same group at different time points and the unpaired t-test to compare the parameters between different groups. Gaussian values were determined using D’Agostino and Pearson for a normality test by the test for datasets of body weight, voiding frequency, voided volume, tactile allodynia and the concurrent determination of abdominal withdrawal response and grimace score utilizing GraphPad Prism 9 Version 9.2.0. In parallel, a repeated-measures 2-way ANOVA was used to determine the differences between the groups over time and treatments utilizing GraphPad Prism 9 Version 9.2.0. Data with *p* ≤ 0.05 difference were considered statistically significant. Data with *p* ≤ 0.05 difference were considered statistically significant.

## 3. Results

### 3.1. Repeated Low-Dose Acrolein-Induced Irreversible Lamina Propria Edema in Urinary Bladder

**Figure 1:**
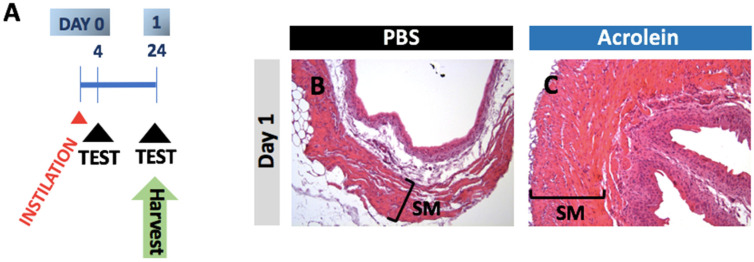
Conventional model of 85 μL acrolein-induced thickened smooth muscle in urinary bladder. (**A**) Experimental design to study the effects of a one-time transurethral instillation of 85 μL acrolein in the urinary bladder of adult C57BL6/J mice. (**B**,**C**) Representative images of hematoxylin and eosin (H&E)-stained images of the urinary bladder of mice in the PBS and acrolein groups.

**Figure 2:**
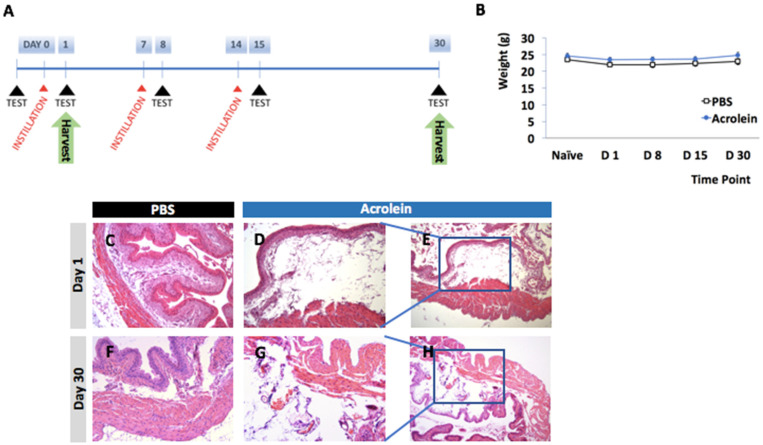
Murine model of a repeated low-dose of acrolein-induced lamina propria in urinary bladder. (**A**) Experimental design to study the effects of the three-time transurethral instillation of 10 μg acrolein and 15-day treatment-free period on the urinary bladder of adult C57BL6/J mice. (**B**) Changes in body weight of mice in the PBS group (PBS mice) and acrolein group (acrolein group). (**C**–**H**) Representative images of hematoxylin and eosin (H&E)-stained images of the urinary bladders of PBS and acrolein mice.

To demonstrate our ability to recapitulate an existing model of acrolein-induced cystitis, we first performed a one-time transurethral instillation of 85 μg acrolein in adult female C57BL/6J mice. As previously designed and described by many researchers in the field, the hematoxylin and eosin (H&E) staining of the formalin-fixed paraffin-embedded (FFPE) tissue of urinary bladder was performed 24 h pi (Figure 1). The tissue of urinary bladder was harvested 24 h pi and processed for formalin-fixed paraffin-embedded (FFPE) tissue. The thickened smooth muscle of the urinary bladder at 24 h pi was shown. After we determined our ability to recapitulate a one-time high dose of acrolein-induced histopathological abnormality in a urinary bladder, we established a murine model of repeated low-dose acrolein by instilling 10 μg acrolein three times transurethrally in adult female C57BL/6J mice as shown in Figure 2A. The repeated low-dose acrolein instillation did not alter significant changes in body weight (Figure 2B). The analysis of all datasets of body weight from PBS vs. acrolein group, at each time, was shown not to reject the hypothesis of normality as determined by the D’Agostino and Pearson test. The subsequent analysis of these data revealed no significant effects as determined by a repeated-measures two-way ANOVA. Lamina propria edema in urinary bladder was observed in acrolein mice on day 1 pi and this histopathological abnormality was irreversible until day 30 pi, which is the time point after 15 days of the acrolein instillation-free period (Figure 2C).

### 3.2. Transient Voiding: High Volume Low Frequency (HVLF)

**Figure 3:**
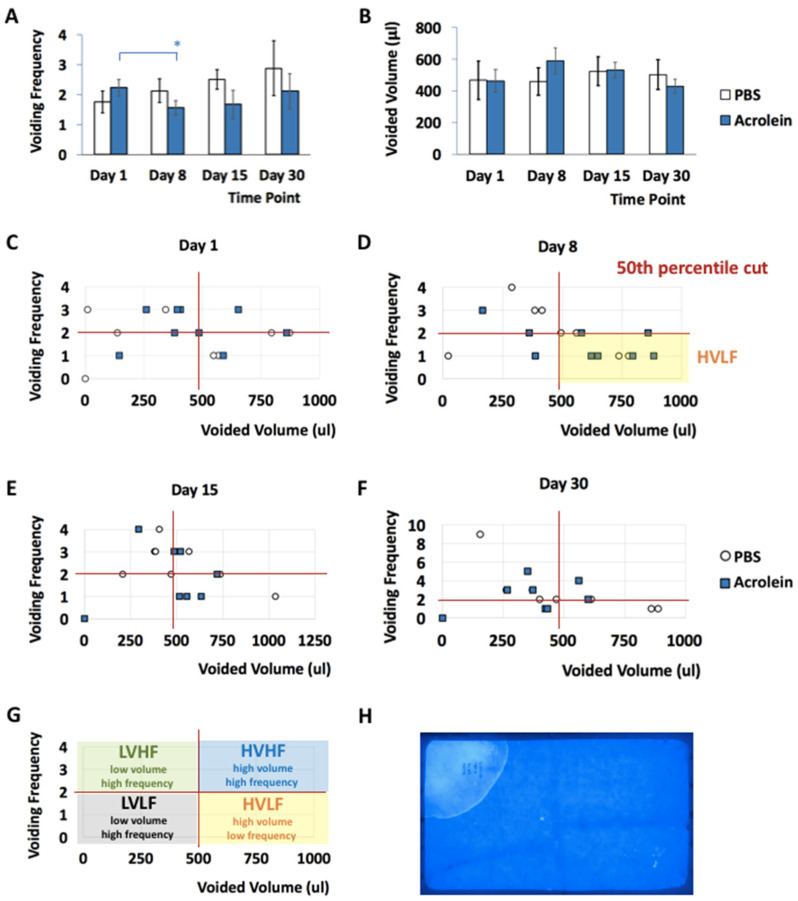
Micturition patterns in PBS and acrolein mice evaluated by filter paper assay (FPA). (**A**) The number of urine spots (≥1.0 cm) in PBS and acrolein mice. * *p* ≤ 0.05 to respective acrolein group values on day 1 pi. (**B**) Analysis of voided volume per void (μL) of the same groups of animals. 2D plot of the voiding frequency and volume per void (μL) for each PBS and acrolein mouse at days 1 (**C**), 8 (**D**), 15 (**E**) and 30 (**F**) having a 50th percentile cut-off point. (**G**) Exemplary plot of voiding frequency and voided volume per void (μL) with the domain of low volume high frequency (LVHF), high volume high frequency (HVHF), low volume high frequency (LVHF) and high volume low frequency (HVLF) defined by the presented percentile cut of frequency and volume. (**H**) Exemplary image of a filter paper scanned under UV light.

Voiding patterns of mice in the repeated low-dose of acrolein (acrolein mice) and control groups (PBS mice) were determined by filter paper assay (FPA) as previously described [19,20]. No significant differences in the voiding frequency and voided volume of acrolein vs. PBS mice were shown at days 1, 8, 15 and 30 pi (Figure 3A,B). The analysis of all datasets of voiding frequency and voided volume revealed no rejection of the hypothesis of normality from the PBS vs. acrolein group at each time point, with the exception of the 30-day pi PBS group. Though this one comparison appears to suggest a deviation from normality, this single event was considered to be an outlier and the hypothesis of normality accepted for that group as well. No significant effects were noted as determined by the repeated-measures two-way ANOVA. Notably, the voiding frequency of acrolein mice at day 8 pi was significantly lower than the voiding frequency of acrolein mice at day 1 pi. The voiding patterns of acrolein vs. PBS mice were categorized as low volume high frequency (LVHF), high volume high frequency (HVHF), low volume high frequency (LVHF) and high volume low frequency (HVLF) based on the 50th percentile cut (Figure 3C–G). On day 8 pi, a higher number of acrolein mice showed a HVLF voiding pattern as compared to PBS mice.

### 3.3. Widening of Eyes in Response to Mechanical Stimuli

**Figure 4:**
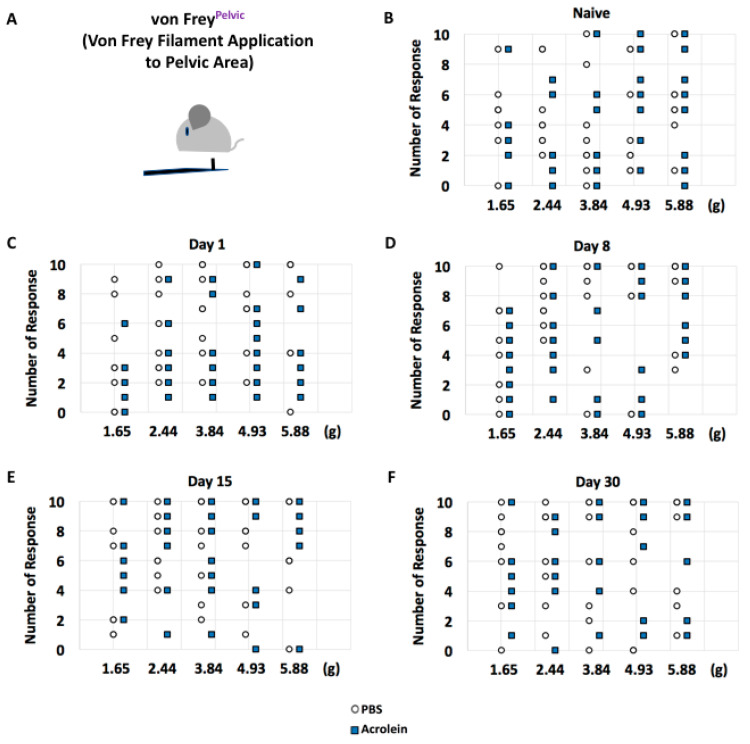
Assessment of abdominal withdrawal response to tactile stimulation in PBS vs. acrolein mice on the time points of naïve, days 1, 8, 15 and 30 pi as determined by von Frey application to the pelvic area. (**A**) Description of von Frey application to the pelvic area. The number of responses in response to an application of von Frey filament 1.65, 2.44, 3.84, 4.92 and 5.88 at naïve, days (**B**), 1 (**C**), 8 (**D**), 15 (**E**) and 30 (**F**) pi.

To determine whether repeated low-dose acrolein triggered scored symptoms in mice, we adapted six methods which were designed to determine scored symptoms in mice. A 10-times application of von Frey filaments revealed no significant difference in the median value of the number of responses in the acrolein vs. PBS group at days 1, 8, 15 and 30 pi (Figure 4).

**Figure 5:**
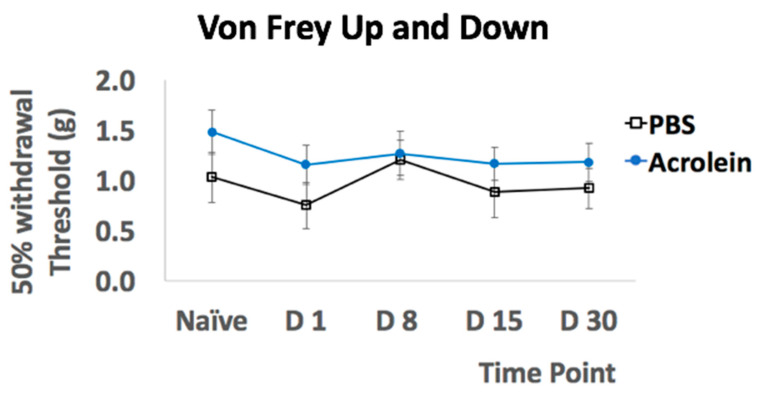
Assessment of mechanical allodynia presented by a 50% withdrawal threshold.

An assessment to determine tactile allodynia of hind paw by the von Frey filaments performed through Dixon’s up–down method presented by 50% withdrawal (Figure 5) did not show any significant difference between the PBS and acrolein group. The hypothesis of normality was not rejected with all the datasets of tactile allodynia from PBS vs. acrolein group at each time point. No significant differences were determined by the repeated-measures two-way ANOVA.

Grimace scale to determine a facial expression of pain did not reveal any significant difference. The fourth behavioral assessment was to evaluate the behavioral status of (1) placing and stepping; (2) righting reflex; (3) spinal symmetry; (4) symmetric ablation; (5) lean; (6) hunched back; (7) catalepsy; (8) tremor; and (9) seizures by using the daily parameter score system (Supplementary Figure S1). In addition to this assessment, up to four mice in either the PBS and acrolein group were scored for (1) placing and stepping; (2) leaning; and (3) having a hunched back. None of the mice in the PBS and acrolein groups were scored “1” and “2” with regard to the behavioral status of (1) righting reflex; (2) spinal symmetry; (3) symmetric ablation; (4) catalepsy; (5) tremor; and (6) seizures (Supplementary Figure S2).

**Figure 6:**
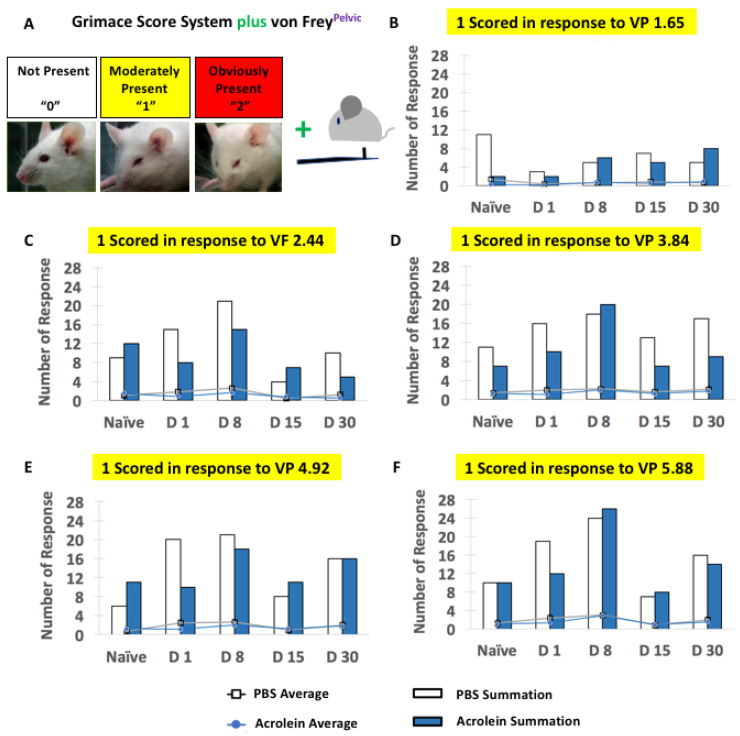
Concurrent assessment of the facial expression of pain and pelvic sensitivity shown in PBS vs. acrolein mice on the time points of naïve, days 1, 8, 15 and 30 pi as determined by the grimace scoring system and the application of von Frey filaments to the pelvic area. (**A**) Description of the grimace scoring system with a combination of the application of von Frey filaments to the pelvic area. The number of responses (bar graphs) and mice that scored 1 in response to the application of the von Frey filament were 1.65 (**B**), 2.44 (**C**), 3.84 (**D**), 4.92 (**E**) and 5.88 (**F**).

The concurrent determination of the abdominal withdrawal response to the tactile stimulation and grimace score revealed that no significance exists in the number of mice that scored either a “1” or “2” based on the grimace scale in response to von Frey filament application to the pelvic area between the PBS vs. acrolein group (Figure 6 and Figure S3). Interestingly, a higher number of mice in both the PBS and acrolein groups were scored “1” rather than “2” in response to the von Frey filament application to the pelvic area between the PBS vs. acrolein groups. Most datasets of the concurrent determination of abdominal withdrawal response to tactile stimulation and grimace score from PBS vs. the acrolein group at each time point passed the normality test as determined by the D’Agostino and Pearson test. Exceptions exist among some datasets and it can be assumed that the superior results based on the results of the D’Agostino and Pearson test for other datasets from the same animals in this study suggest that the same underlying distribution occurred under same treatment conditions in this study. The repeated-measures two-way ANOVA revealed the time-dependent significance of the group of animals that were one scored VP 2.44 (Figure 6C), 1scored VP 4.93 (Figure 6E) and one scored VP 5.88 (Figure 6F). No significant differences were determined by the repeated-measures two-way ANOVA in Supplementary Figure S3.

**Figure 7:**
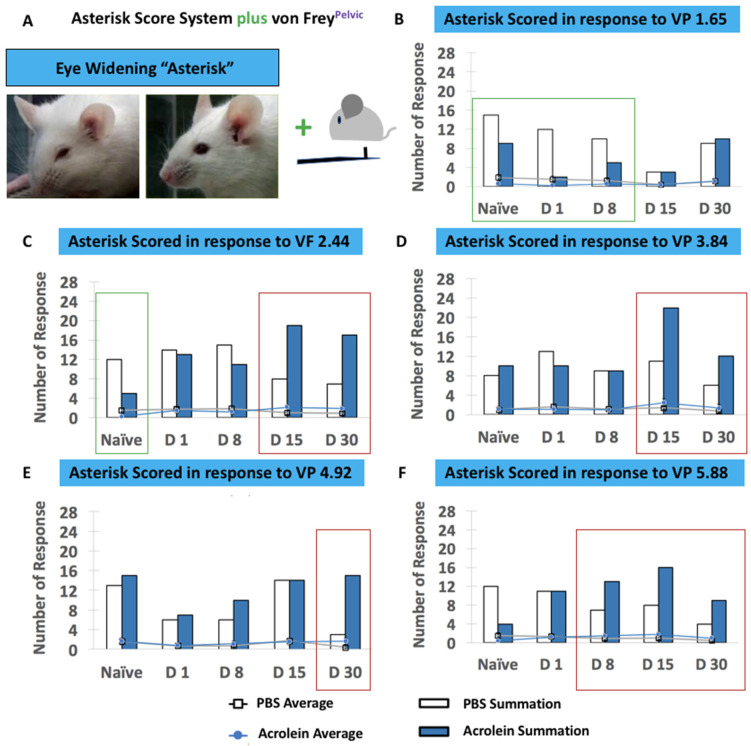
Concurrent assessment of the facial expression of pain and pelvic sensitivity shown in PBS vs. acrolein mice on the time points of naïve, days 1, 8, 15 and 30 pi as determined by the asterisk scoring system and von Frey application to the pelvic area. (**A**) Description of the asterisk scoring system with a combination of the application of von Frey filaments to pelvic area. The number of responses (bar graphs) and mice that scored an asterisk in response to the application of von Frey filaments 1.65 (**B**), 2.44 (**C**), 3.84 (**D**), 4.92 (**E**) and 5.88 (**F**).

While we performed this concurrent assessment, we identified a new behavioral phenotype of the widening of the eyes upon the application of von Frey filaments which was not recognized by the grimace score system, and this was scored as an asterisk * (Figure 7A). Interestingly, a higher number of mice in the PBS group were asterisk scored in response to the von Frey filament 1.65 at naïve, days 1 and 8 pi (green box, Figure 7B) as well as the von Frey filament 2.44 at naïve, (green box, Figure 7C). In contrast, more mice in the acrolein group were asterisk scored in response to thicker von Frey filaments at later time points (red box, Figure 7C–F). No significant difference was determined by repeated-measures two-way ANOVA in Figure 7.

**Figure 8:**
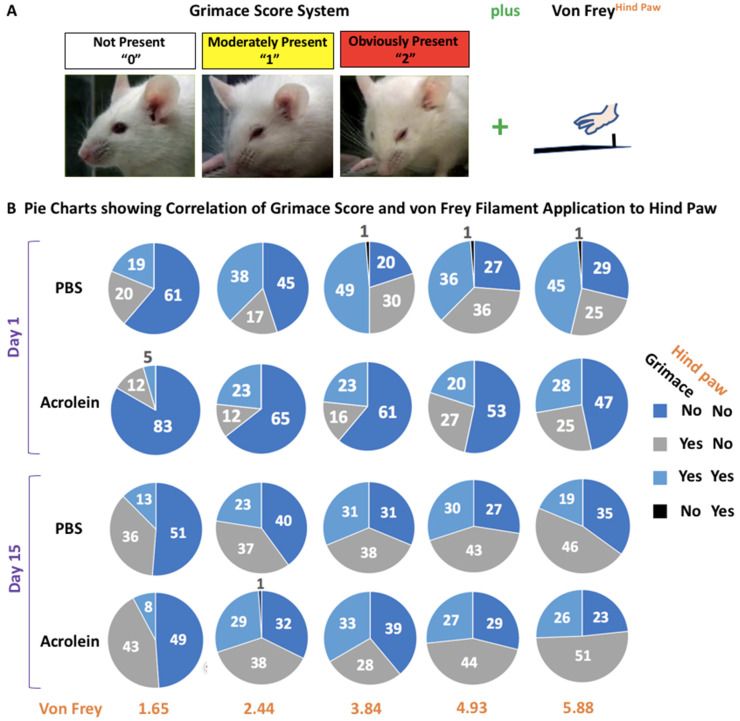
Facial expression of pain and peripheral sensitivity determined by the grimace scoring system and von Frey application to the hind paw. (**A**) Description of the grimace scoring system with a combination of von Frey application to the hind paw. (**B**) Pie charts showing the percentage (%) of mice determined to be neither grimace scored nor responsive to von Frey filaments (dark blue), to be grimace scored but not be responsive to von Frey filaments (gray), to be grimace scored and responsive to von Frey filaments (light blue), and to be not grimace scored but responsive to von Frey filaments (black) on days 1 and 15.

The experimental overview and results of the concurrent assessment of the tactile allodynia of the hind paw and grimace score are shown in Figure 8. At day 1 pi, a higher % of acrolein mice were scored a “1” or “2” by the grimace scale and were responsive to von Frey filament application to the hind paw as compared to a % of PBS mice (blue in pie chart, yes for grimace/yes for von Frey). Interestingly, this difference was not shown at day 15 pi.

**Figure 9:**
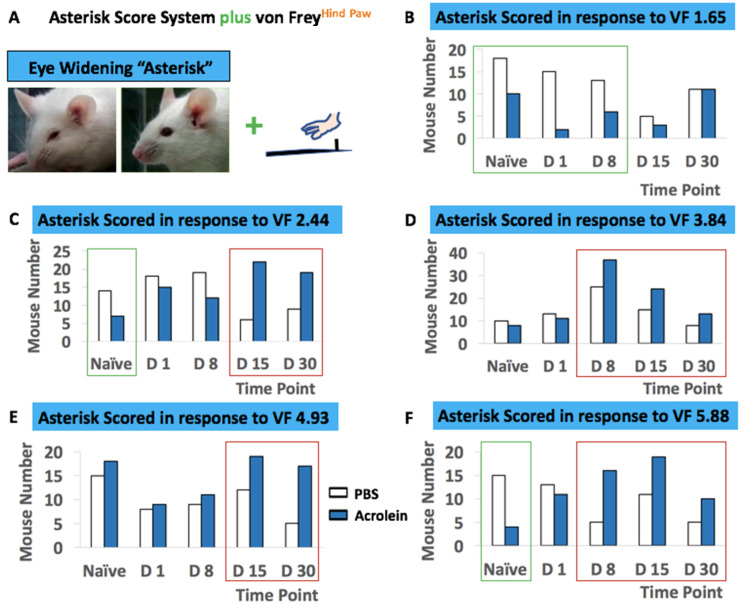
Concurrent assessment of the facial expression of pain and mechanical allodynia shown in PBS vs. acrolein mice on the time points of naïve, days 1, 8, 15 and 30 as determined by the asterisk scoring system and the application of von Frey filaments to the hind paw. (**A**) Description of the asterisk scoring system with a combination of von Frey application to the pelvic area. The number of responses (bar graphs) and mice that scored an asterisk in response to the application of von Frey filaments 1.65 (**B**), 2.44 (**C**), 3.84 (**D**), 4.92 (graph says 4.93, should be 4.92) (**E)** and 5.88 (**F**).

While we performed our last behavioral assessment, which was a concurrent determination of tactile allodynia of the hind paw and grimace score, we also identified the behavioral phenotype of the widening of the eyes upon the von Frey filament application (Figure 9A). As this pattern was observed in Figure 7, more mice in the PBS group were asterisk scored in response to the von Frey filament 1.65 at naïve, days 1 and 8 pi (green box, Figure 9B) as well as the von Frey filaments 2.44 and 5.88 at naïve (green box, Figure 9C,F). Up to four times more mice in the acrolein group were asterisk scored in response to von Frey filaments 2.44 and 4.93 at days 15 and 30 pi (red box, Figure 9C,E). Likewise, up to three times more mice in the acrolein group were asterisk scored in response to von Frey filaments 3.84 and 5.88 at days 8, 15 and 30 pi (red box, Figure 9D,F).

## 4. Discussion

Repeated low-dose acrolein triggered irreversible histopathological changes in mouse urinary bladder and transient voiding behavior, which is different from the acute cystitis model. Furthermore, we demonstrated that no significant difference in PBS vs. acrolein mice was observed, as determined by the conventional assessment of pain, von Frey application method and the grimace score system. Our new scoring criteria for the facial expression of the widening of eyes revealed that the tendency was that more PBS mice were asterisk scored in response to lighter von Frey filaments at earlier time points vs. more acrolein mice that were asterisk scored in response to heavier von Frey filaments at later time points.

Since a substantial number of patients receiving CYP treatment are known to undergo repeated periodic exposures, our murine model was designed with three repeated treatments at an interval of 7 days each. Our histopathological assessment of H&E staining of the urinary bladder revealed lamina propria edema and this was not reversible even after two weeks of an acrolein treatment-free period. This was consistent with the clinical observation that CYP-induced histopathological changes that were persistent in patients, as determined by repeated cystoscopic evaluations [4].

Our study suggests that urinary symptoms among human subjects having undergone prolonged low-dose exposure to acrolein may differ from previously reported urinary symptoms among patients that have received a high dose of acrolein for their therapy. Our observation is consistent with a previous study that CYP-induced nephrotoxicity caused polyuria, meaning an abnormally large volume of urination per urinary episode, in a rat model [27].

To determine the role of repeated low-dose acrolein on scored symptoms, we adapted six different assessments: (1) abdominal withdrawal response; (2) tactile allodynia; (3) grimace score; (4) daily parameter; (5) concurrent determination of abdominal withdrawal response to tactile stimulation and grimace score; and (6) concurrent determination of tactile allodynia of hind paw and grimace score. These assessments can be categorized by their experimental methods into the von Frey method, grimace scoring system and experimental intervention to merge the von Frey method and grimace scoring system. Here, von Frey filaments were used to determine tactile allodynia. Abdominal withdrawal response to tactile stimulation was determined by the up–down method [26,28,29,30] and the 10-times application method [31,32,33]. Given that the tactile allodynia of hind paw and the abdominal withdrawal response to tactile stimulation are a common consequence of being in a state of pain, these methods have been widely used to study whether pain is modulated in the area of interest in research. Particularly for bladder health, the correlation and causality among bladder physiology, voiding function and the modulation of the pain mechanism have been widely studied [34]. This can be explained by the pathophysiological features of the urinary bladder coordinating activities of the nervous systems, smooth muscle, urothelium and other components. In that sense, pathophysiological modulation in the urinary bladder the alters properties of afferent sensory pathways resulting in differential responses to mechanical stimuli [35] which can be determined by the von Frey method. In parallel with the von Frey method, the grimace score system has been introduced in mouse [22,23], rat [36] and horse models [37] as a tool to assess the pain level. The grimace score system is expected to capture spontaneous and chronic pain as compared to the von Frey method which measures the withdrawal response to mechanical stimuli [36]. In our study, acrolein-induced abnormal histopathology in the urinary bladder does not trigger tactile allodynia, abdominal withdrawal response to tactile stimulation or differential facial expression in adult C57BL6/J mouse. This may suggest a need to determine whether patients who develop CYP-induced abnormal histopathology in the urinary bladder may not have distinct symptoms to recognize CYP treatment side effects. Conversely, CYP may not trigger histopathological changes in the urinary bladder but may produce symptoms previously reported by this population of patients [4]. Taken together, the polymorphic phenotype of CYP-induced side effects and its prolonged persistence emphasizes that a symptom-based assessment of CYP-induced hemorrhagic cystitis is required but there is also a need to follow up and monitor patients who undergo CYP treatment regardless of symptom existence. In parallel, the scoring of bladder pathologies and its correlation with the symptom scoring may suggest further insights regarding the existence of an inverse relation to systemic allodynia and its local effects in the bladder. Furthermore, the characterization of the cellular phenotype responsible for the edema of the lamina propria and the thickening of smooth muscle may suggest a better understanding of its cellular mechanism.

In addition to the two conventional methods that determine pain and score symptoms, we merged these two methods by performing a concurrent assessment to determine the differential changes in facial expressions of pain in response to mechanical stimuli. During this process, we identified a new behavioral change of widening eyes and labelled this behavior as an asterisk score. There was a tendency of the PBS animal group to have a higher asterisk score in response to mechanical stimuli at an earlier stage but the acrolein animal group had a higher asterisk score in the later stage of the experimental timeline, which may suggest the increased recognition and sensation of mechanical stimuli to periphery, but may not result in withdrawal response.

The clinical implication of the widening of eyes to mechanical stimuli that it indicates either acute, transitional or chronic pain status should be further determined by the characterization of neuronal, immune or neuro-immune mechanisms as well as other mechanistic targets [38,39] in asterisk-scored animals. On the other hand, our study also revealed that the reliability of the scoring system remains limited in a murine model.

In the study reported by Stillwell et al., 28 patients were pediatric among the 100 patients that developed CYP-induced cystitis [4]. Furthermore, lower doses and shorter durations of intravenous CYP treatment produced cystitis in pediatric patients as compared to adult patients. This suggests the compounding role that CYP/acrolein has on the bladder health of pediatric patients, however, the majority of murine models studying CYP-induced cystitis were established in the adult mouse. On the other hand, it has been discussed that 82% of pediatric patients who had CYP-induced hemorrhagic cystitis received CYP parenterally, suggesting that the route of CYP treatment may matter more than the treatment age for the cause of hemorrhagic cystitis [4].

## 5. Conclusions

Repeated low doses of acrolein trigger irreversible lamina propria in urinary bladder, high volume low frequency voiding and a differential facial expression of widening eyes in response to mechanical stimuli.

## Data Availability

All data analyzed during this study are included in this published article and its supplementary information files.

## Supplementary Materials

The following are available online at https://www.mdpi.com/article/10.3390/cells10123477/s1, Figure S1: Assessment of facial expression of pain shown in PBS vs. Acrolein mice on the time points of Naïve, day 1, 8, 15 and 30 by the Asterisk scoring system, Figure S2: Evaluation of behavioral status of PBS vs. Acrolein mice on the time points of Naïve, Figure S3: Concurrent assessment of facial expression of pain and pelvic sensitivity shown in PBS vs. Acrolein mice on time points of Naïve.
