# Supplementary material for: Repeated Low-Dose Acrolein Triggers Irreversible Lamina Propria Edema in Urinary Bladder, Transient Voiding Behavior and Widening of Eyes to Mechanical Stimuli
Source: Cells. 2021 Dec 9;10(12):3477. doi: 10.3390/cells10123477 (PMC8700116; doi:10.3390/cells10123477)
Supplement: Supplementary file 1 [file cells-10-03477-s001.zip › cells-1389893-supplementary.pdf]

Supplementary Information

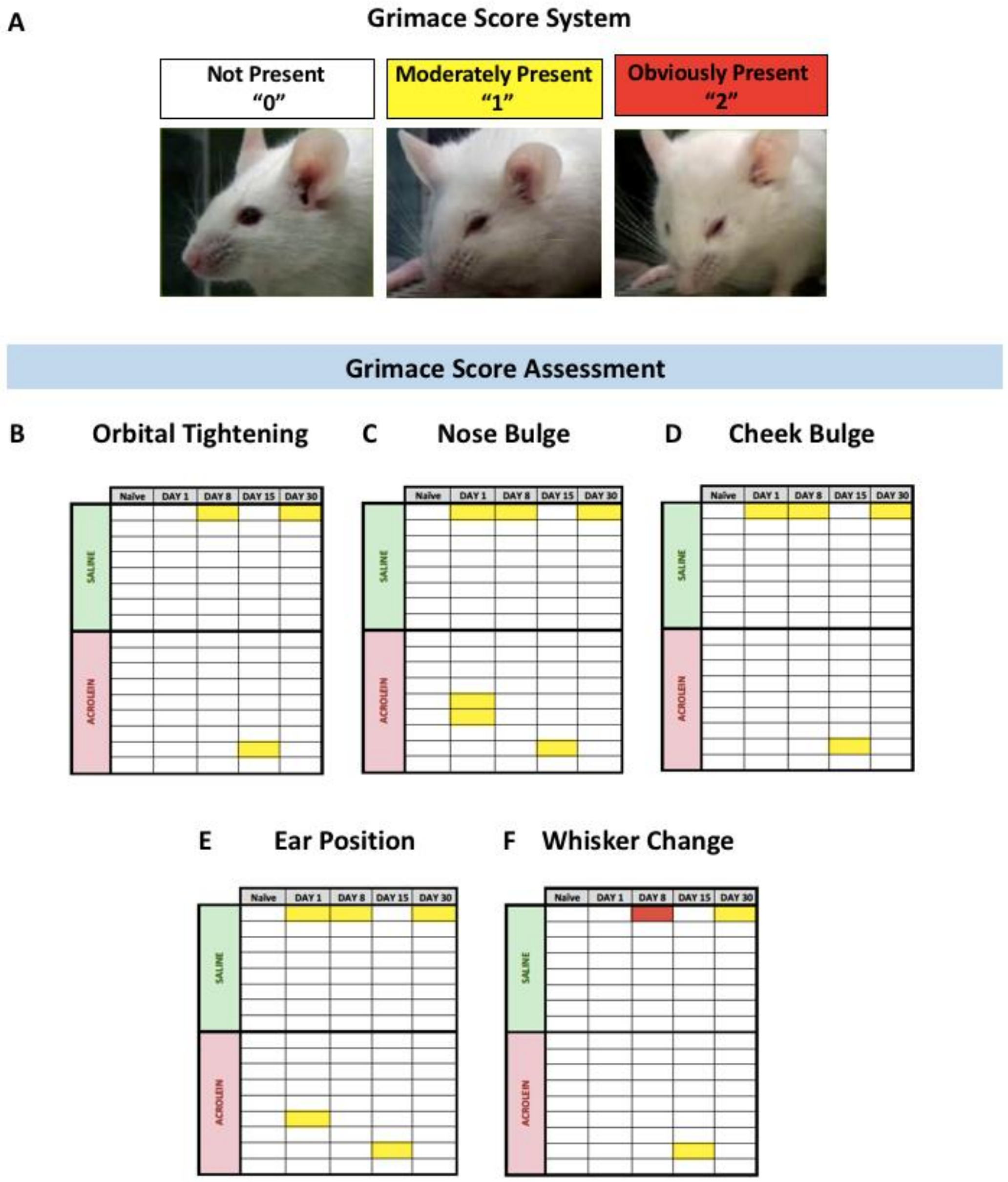

Figure S1. Assessment of facial expression of pain shown in PBS vs Acrolein mice on the time points of Naïve, day 1, 8, 15 and 30 by the Asterisk scoring system. (A) Scale Description of Grimace scoring system. Colorimetric presentation of Grimace score for each mouse based on the assessment criteria of orbital tightening (B), nose bulge (C), cheek bulge, (D) ear position (E) and whisker change (F).

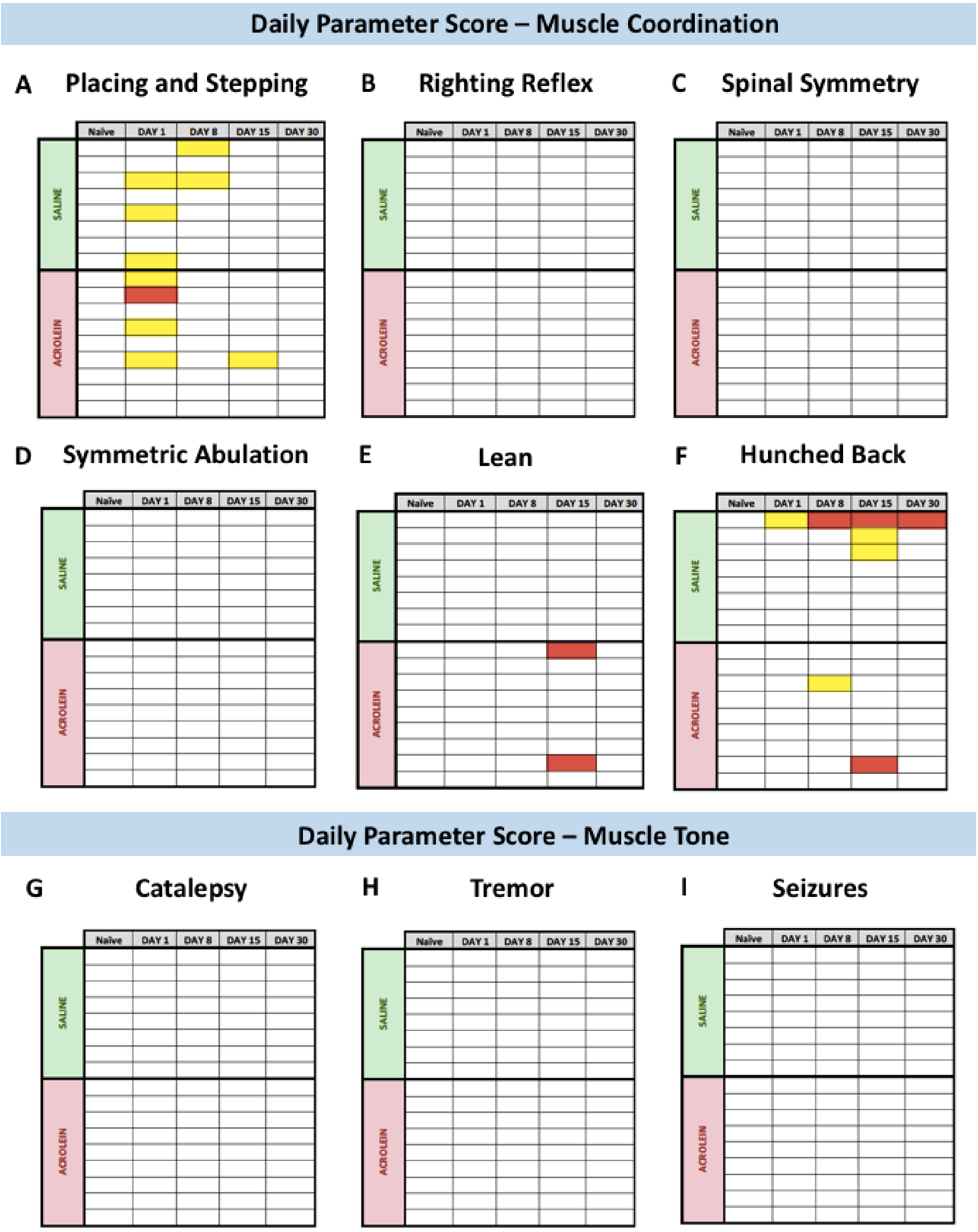

Figure S2. Evaluation of behavioral status of PBS vs Acrolein mice on the time points of Naïve, day 1, 8, 15 and 30 pi by using a daily parameter score system on the following scale: white = normal with no clinical signs, yellow = mild to moderate clinical signs, and red = severe clinical signs. Colorimetric presentation of behavioral status of each mouse based on the assessment criteria of placing and stepping (A), righting reflex (B), spinal symmetry (C), symmetric ambulation (D), lean (E), hunched back (F), catalepsy (G), tremor (H) and seizures (I).

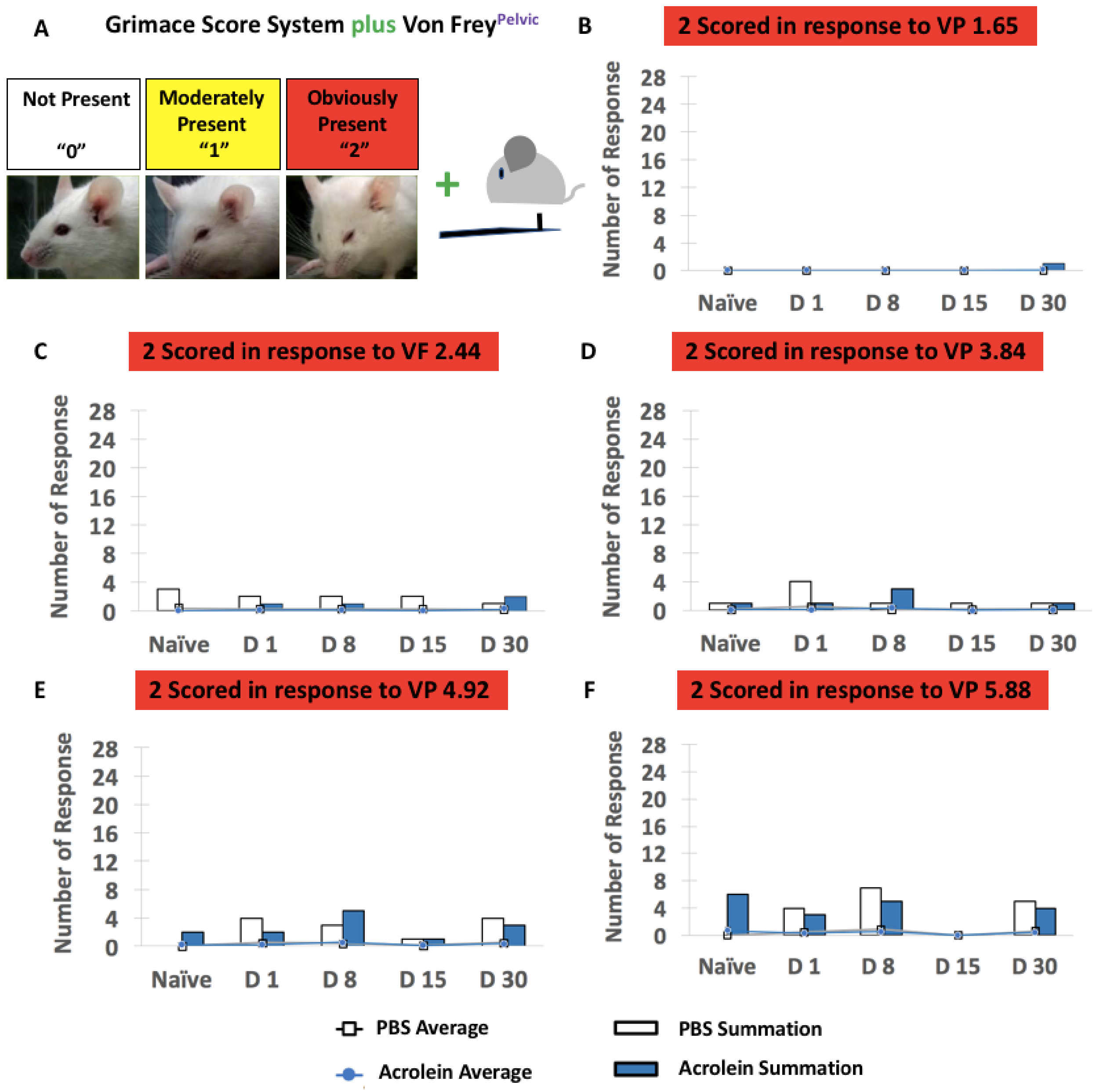

Figure S3. Concurrent assessment of facial expression of pain and pelvic sensitivity shown in PBS vs Acrolein mice on time points of Naïve, day 1, 8, 15 and 30 pi as determined by the Grimace scoring system and Von Frey application to the pelvic area. (A) Description of the Grimace scoring system with a combination of Von Frey application to the pelvic area. The number of responses (bar graphs) and mice that scored 2 in response to the application of Von Frey filament 1.65 (B), 2.44 (C), 3.84 (D), 4.92 (E) and 5.88 (F).
